# Reaction Force/Torque Sensing in a Master-Slave Robot System without Mechanical Sensors

**DOI:** 10.3390/s100807134

**Published:** 2010-07-29

**Authors:** Tao Liu, Chunguang Li, Yoshio Inoue, Kyoko Shibata

**Affiliations:** Department of Intelligent Mechanical Systems Engineering, Kochi University of Technology, 185 Miyanokuchi, Tosayamada-Cho, Kami-City, Kochi 782-8502, Japan; E-Mails: 126011u@gs.kochi-tech.ac.jp (C.L.); inoue.yoshio@kochi-tech.ac.jp (Y.I.); shibata.kyoko@kochi-tech.ac.jp (K.S.)

**Keywords:** force sensing, master-slave robot, mirror-image movement, reaction force

## Abstract

In human-robot cooperative control systems, force feedback is often necessary in order to achieve high precision and high stability. Usually, traditional robot assistant systems implement force feedback using force/torque sensors. However, it is difficult to directly mount a mechanical force sensor on some working terminals, such as in applications of minimally invasive robotic surgery, micromanipulation, or in working environments exposed to radiation or high temperature. We propose a novel force sensing mechanism for implementing force feedback in a master-slave robot system with no mechanical sensors. The system consists of two identical electro-motors with the master motor powering the slave motor to interact with the environment. A bimanual coordinated training platform using the new force sensing mechanism was developed and the system was verified in experiments. Results confirm that the proposed mechanism is capable of achieving bilateral force sensing and mirror-image movements of two terminals in two reverse control directions.

## 1. Introduction

Many kinds of assistant robots have been developed to help human operators implement complex tasks in different fields of application. For instance, rehabilitation robots [1–6] can deliver motor recovery therapy by delivering a suitable force to hemiplegic patients during training. Thus, the workload of therapists and the economic burden of society and patients can be reduced to a certain extent. It has also been confirmed that robot-assisted rehabilitation training can produce more encouraging results than conventional therapy provided by therapists alone. Surgical robots [7–10] can assist surgeons in finishing operations with high accuracy and safety. In addition, assistant robots make it possible to carry out minimally invasive surgery that is difficult to implement by human surgeons. Some remote robots (for instance, working in an isolation room) can aid operators in performing operations in extreme environments [11–13] with high temperature or radiation. In addition, many micro-manipulations [14–18] can be implemented with the aid of assistant robots. In robot assistant systems, human operators cooperate with robots, thus force feedback/sensing is necessary to assure system stability and safety. Based on force feedback/sensation, operators can regulate the control/input force accordingly, to further reduce the pain suffered by patients during the process of rehabilitation training, or mis-operations in surgery and other kinds of manipulations.

The driver SEAT system [19] is a self-assisted device supporting bilateral steering training. Subjects can perform bilateral steering tasks in a driving simulation environment with active force-feedback cues. In order to increase the productive use of a patient’s impaired arm, a stiffening of the wheel in proportion to the healthy arm’s use is considered as a force feedback cue, to provide a reminder when the healthy arm is being overused. Experimental results have verified that the force cues had a positive effect on increasing the productive torque activity of the impaired arm. This was also confirmed by the increased EMG activity in several muscles of the impaired arm. Park and Peng [20,21] presented a portable tele-rehabilitation system for the treatment and assessment of elbow deformity of stroke patients. A real-time control strategy and a teach-and-replay control method are achieved for tasks involving slow movements and fast movements, respectively. The torque and position control modes for the master and slave devices can be exchanged for passive and active movements. Thus the system supports both passive and active movements including slow and fast tasks. For both slow and fast movements, transparent haptic feeling enables clinicians to give a correct assessment of the motion capability of patients and to regulate the training strategy properly. Guo and Song [22] introduced a VR-based rehabilitation system to support self-assisted training for mild stroke patients. Two hands are coordinated to control a virtual stick to move across a predefined route that displayed in a personal computer. The injured and healthy hands control the position of the stylus of a PHANTOM haptic device and the pose of an MTx inertial sensor, respectively. The pose of the MTx inertial sensor includes roll and pitch in two degrees of freedom. The angles of roll and pitch determine the angle of the virtual stick and the corresponding force exerted on the injured hand, respectively. Thus, patients can change the difficulty of training tasks by adjusting the pitch angle and rotate the virtual stick by altering the roll angle. Furthermore, the healthy hand can assist the injured hand in the accomplishment of tasks at different levels of difficulty.

However, the assistant robots described above realize force feedback/sensing by using force sensors or complex impedance controllers. As a result, system cost, hardware mounting difficulty, and spatial requirements are increased somewhat. Our previous work has introduced a master-slave robotic prototype to implement force sensing without using any force sensor or impedance/force controller [23,24]. The system realized master-slave mirror-symmetric movements, which is an essential requirement for performing various operations in robotic systems designed for rehabilitation [25–28], medical operation [29], remote control [30], and so on. Preliminary experiments have verified the feasibility of the novel force sensing mechanism. However, the system can not be used in applications requiring a relatively large driving force due to its limited driving torque. This paper presents an improved master-slave device with a larger driving torque to support bilateral arm cooperative training. Except for a further verification of the force sensing performance, frequency response range and the sensing capability in resistant and assistant forces were also confirmed.

## 2. Working Mechanism

**Figure 1.**
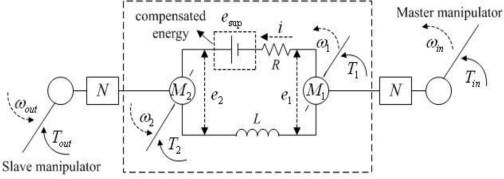
Equivalent circuit of the master-slave control system.

As shown within the dashed wire frame in Figure 1, a master-slave system consists of two identical DC motors connected directly to construct a closed-loop circuit. One motor behaves as a generator (master motor: *M*_1_) and powers the other (slave motor: *M_2_*), which works in an electro-motive state and supports an end-effecter to accomplish various operations. Hence, a kind of energy recycling is achieved. The two motors have identical electromagnetic torques (*T_M_*) because of the shared closed-loop current and the same motor torque constant. Therefore, their mechanical torques are connected with each other by the current. That is, the torque variation in one motor shaft can be reflected to the contra-lateral side. Then, the operator adjusts the control force accordingly based on the sensed force to achieve a balanced torque state. Thereby, the system realizes force sensing without using a force sensor. In addition, the force sensing mechanism (closed-loop current) make the system have bidirectional controllability.

Considering the analysis in [24], the relationship between mechanical torques in the two motor shafts can be expressed as:

(1) $$T_{1}=T_{2}+2T_{0}+J_{1}\frac{d\omega_{1}}{\mathrm{dt}}+J_{2}\frac{d\omega_{2}}{\mathrm{dt}}$$

The unloaded torques (*T_0_*) and inertial torques ( $J\frac{d\omega }{\mathrm{dt}}$) of the motors are negligible because they are very small compared to the mechanical torques in the motor shafts (*T_1_* and *T_2_*). In some applications, a high driving torque/force is required to deal with the larger reaction force. In this case, gear boxes can be applied to increase the output torque of the system (Figure 1). Two identical gear boxes are employed to make a symmetric mechanism and to ensure that the system behaves the same in both control directions. The new relationship between terminal torques is:

(2) $$T_{\mathrm{in}}=\frac{N_{1}}{N_{2}}\frac{T_{\mathrm{out}}}{\eta_{1}\eta_{2}}+\frac{N_{1}}{\eta_{1}}(2T_{0}+J_{1}\frac{d\omega_{1}}{\mathrm{dt}}+J_{2}\frac{d\omega_{2}}{\mathrm{dt}})$$

here *N_1_* = *N_2_*. A larger input torque is required to balance the same reaction torque due to the gear efficiency and amplified unloaded and inertial torques of the motors. In this way, motors and gear boxes with high working efficiency are preferable in order to reduce the requirement on input torque. If a significant amount of torque is required for the actuator on the slave side, a longer force arm in the master terminal can be designed to reduce the force requirement for an operator. Referring to Equation (2), the force relationship between the two terminals can also be changed by matching the gear ratios of the gear boxes. The requirement for the input torque/force can be reduced by using a gear box that has a smaller gear ratio on the master side. In addition, the relationship between the torques on the motor shafts can be changed by selecting motors with different torque constants, which can change the relation between the electromagnetic torques of two motors. Thus, the required force in the master terminal can be reduced by using a master motor with a smaller motor torque constant, even though the lengths of force arms and the gear ratios are identical. However, the system will not be symmetric in the two control directions no matter which method is employed to match the terminal forces, whereas it has no influence on unilateral control systems.

In the system, the rotational velocity of the master should be very high to actuate the slave. Otherwise, even though the slave can be rotated with the generated energy of the master, its rotational velocity will be much slower than that of the master due to the energy losses in the resistances and inductances of the two motors *R* and *L*. However, in robot assistant systems, it is always required that the slave terminal reproduces the movement of the master, or that the master and slave have a certain ratio relation in movement trajectories. In order to realize accurate master-slave motion tracking and make it possible to actuate the slave with a slow input velocity, a certain amount of energy *e*_sup_ is compensated for the closed-loop circuit with an H-bridge driver. Based on the velocity and position differences between the two terminals, a motion tracking controller is realized to regulate the control signals (PWM: pulse-width-modulation, and direction) of the H-bridge driver, and further to adjust the amount of compensated energy [24]. The compensated energy, together with the energy generated by the master, assures the motion consistency of the master and slave terminals.

The force sensing mechanism and master-slave closed-loop structure ensure the system has no directional limitations in the configuration of two motors. During operation, the motor attached with a larger torque behaves as the master and the other motor behaves as the slave. Therefore, the system has bidirectional controllability. This is favorable for hemiplegic patients performing bimanual cooperative rehabilitation training, in which the control direction depends on which side is the impaired limb and on the training modes. If the impaired limb is moved passively by the healthy one (passive mode), or it actuates a movement actively but accomplishes movements with a larger assisting force from the healthy one (active-assisted mode), the control direction is from the healthy limb towards the impaired one; if the impaired limb activates the movement and accomplishes the movement with a smaller assisting force (active-assisted mode) or with a relatively small resistant force from the healthy limb (active-resisted mode), the control direction is from the impaired limb towards the healthy one.

In addition, the wired connection between the master and slave motors makes it possible to adjust the relative position of the master and slave according to application requirements. This is an advantage over conventional systems which realize force sensing by connecting the master and slave mechanically [31].

## 3. Experimental Study

### 3.1. Experimental Platform

**Figure 2.**
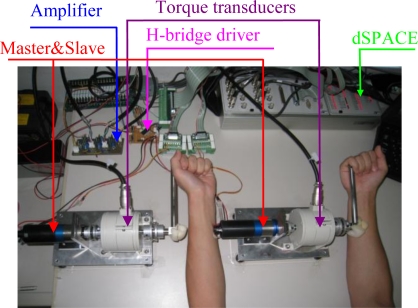
Experimental schematic for bimanual coordinated control.

A preliminary test platform supporting bimanual coordinated upper-limb training was built to verify the effectiveness of the proposed force sensing mechanism (Figure 2). It consisted of master and slave units (motors 3863012C combined with planetary gear boxes 38/2 A and encoders IE2-512, Faulhaber Group, Germany), an H-bridge driver (LMD18200, National Semiconductor, USA), a dSPACE control platform (CLP1104, dSPACE, Germany), two torque transducers (TP-20KCE, Japan), and a torque signal amplifier. The master and slave units were fixed to a height-adjustable and position-adjustable table, the two torque transducers and gear mechanisms were connected coaxially and the two handles were attached to the transducer shafts. Two identical gear boxes with a gear ratio of 66 were employed. The corresponding maximum output torque of the system was 5.082 Nm, which was larger than that of the system presented in [24] and therefore this device is able to support bilateral arm coordinated training. The torque transducers, torque signal amplifier and CLP1104 had the same function with that introduced in [24].

The terminal shafts of the two torque transducers were attached with two isometric handles, which were manipulated by a subject. The device supports passive-active, active-assisted, and active-resisted training modes. The first and second patterns before and after the “-” denote the working states of the weak and strong limbs, respectively. The strong limb will provide a corresponding force for the weak limb in different training modes through this master-slave device. The control direction is defined as the direction from the limb exerting a larger force to the limb imposing a smaller force. It depends on the health conditions of the two limbs and the training modes.

### 3.2. Calibration Test

**Figure 3.**
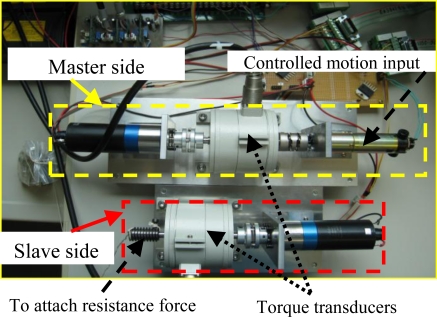
The experimental platform to test the force sensing mechanism in a master-slave system.

In this experiment, the regulation of the input torque during a variation in resistance in the slave terminal was used to verify the force sensing characteristic. A DC driving motor was used to drive the master unit instead of a human operator (Figure 3, two handles removed) in order to simplify the analysis of force sensing performance, because a human operator is unable to rotate the system with a constant velocity. The testing method was same with that presented in previous work [24]. Here the reference velocity was 100 degrees per second. Using a constant value aimed at reducing the frictional/inertial torque variation caused by velocity variation and at testing the force sensing capability with higher accuracy. During the process of rotation, a subject exerted an increased resistance/reference torque on the slave terminal.

**Figure 4.**
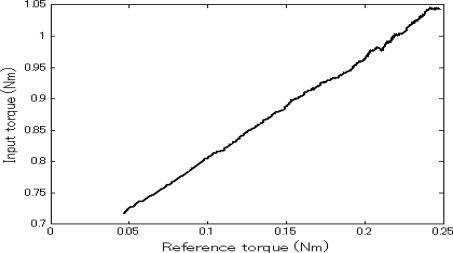
The relationship between the input and output torque.

**Figure 5.**
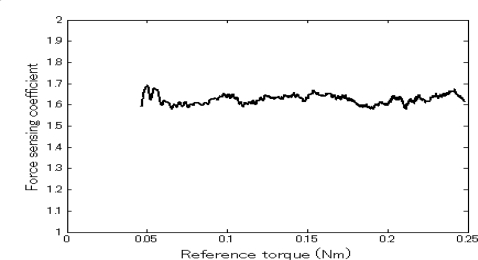
Force sensing coefficient curve.

The force sensing results are shown in Figure 4. It can be seen that the input torque increased with the increment of external resistance/reference torque. This demonstrates the force sensing capability of the system without a force sensor. The corresponding force sensing coefficient (refer to [24]) curve is shown in Figure 5. We can see that the coefficient was approximately constant. The corresponding average value was 1.626. Actually, during the experiment, the master/slave velocity had a small fluctuation around the reference velocity, thus the unloaded and inertial torques of the motors were not constant. That is, the second item in Equation (2) varied slightly. Also, the gear box efficiency was not unchanged under different loads. Therefore, the calculated force sensing coefficient was not a constant. In the experiment, in the velocity varying range of 1.625 degrees per second, the maximum rate of change of the force sensing coefficient is 0.04. Compared to the sensory capacity of a human operator, this fluctuation can be ignored. That is, the force sensing resolution of the system mainly relies on the efficiency of the two gear boxes (refer to Equation (2)), and a value around 1.626 is enough for human operators to sense the variation of the reaction force. Therefore, using the system, an operator can sense the force on the slave side and provide a balancing force on the control side accordingly. Based on the force sensing principle, the force sensing range corresponds to the load-bearing capability of the slave unit, which is 5.082 Nm.

### 3.3. Frequency Response Test

**Figure 6.**
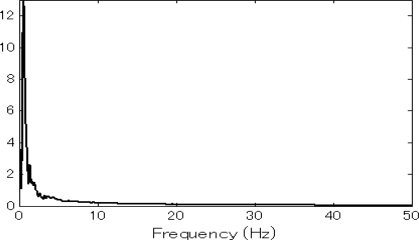
Frequency response curve of the system.

This experiment was also performed by a human operator. In order to test the frequency response range of the system, the operator exerted an increasing force on one side singly and rotated the two handles with an increasing velocity, until the two terminals can not match each other in motion behavior. Then a FFT (Fast Fourier Transform) analysis was carried out with the velocity information collected from the two terminals during the period that the master and slave made mirror symmetric movements. The same FFT results were obtained for the data detected on the master and slave terminals. When the control force was attached to the left unit, the corresponding frequency response is shown in Figure 6, and the same result was obtained for the case when the control force was exerted on the right hand side. This demonstrates that the system can respond to an input signal within the velocity frequency range of 30 Hz, which is sufficient for responding to the control commands of human operators.

### 3.4. Resistant and Assistant Force Sensing Test

**Figure 7.**
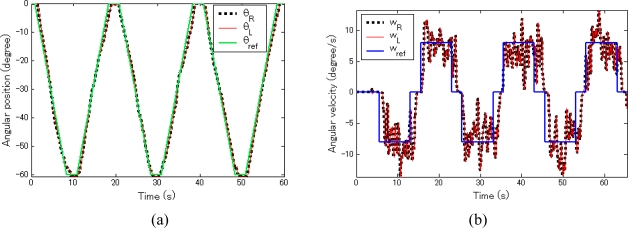
A representative motion tracking trajectory and the corresponding velocity curve. **(a)** Motion tracking trajectory. **(b)** Velocity curve.

The experiment was aimed at verifying that both a resistant force and an assistant force can be sensed by an operator with the system and confirming that the system is feasible to support bimanual training. An operator controlled the force acting on both terminals with two hands and drove the handles to accomplish a predefined dynamic movement (upward and downward: elbow flexion and extension) with a velocity of 8 degrees per second. The test was carried out in two steps: firstly, the left limb provided an active force while the right limb exerted a resistant force (active-resisted mode); secondly, the left hand provided a small force and the right hand exerted an assistant force (active-assisted mode). For the both cases, the same motion tracking trajectory was performed. A representative motion tracking trajectory and the corresponding velocity curve are shown in Figure 7.

**Figure 8.**
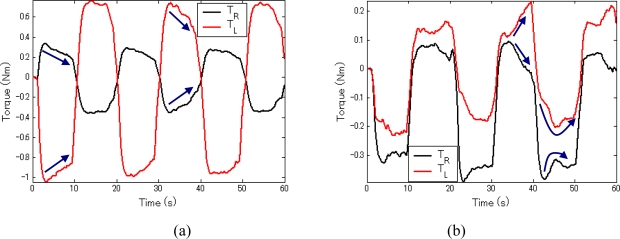
Torque curves in the two terminals. **(a)** Active-resisted mode. **(b)** Active-assisted mode.

The torque curves in the two terminals are given in Figure 8. The subscripts *L* and *R* denote the corresponding parameters in the left and right hand sides, respectively. The master and slave terminals realized symmetric movement accurately with the position and velocity errors between the two terminals of 0.0559 degree and 0.5681 degree per second for the resistant force test, and of 0.0613 degree and 0.5926 degree per second for the assistant force test. In this test, the velocity of 8 degrees per second was low, it is demonstrated that the system is capable of force sensing and movement reproducing for both high (experiment 1) and low velocities. Besides, it can be concluded that the slave can reproduce the master’s movements accurately. Comparing the Figure 8 (a,b), it can be concluded that the resistant force increased the burden on the left hand, while the assistant force reduced the force requirement for the left hand. And the force of the left hand was regulated following the variation of the assistant/resistant force in the contra-lateral side: it had the same varying trend with the resistant force and the reverse varying trend with the assistant force. The results confirm that the operator can sense both a resistant force and an assistant force with the system.

## 4. Discussion

This paper presented a bimanual training device to confirm the feasibility of the force sensing mechanism thoroughly. Compared to the previous work [24], this work demonstrated that the force sensing mechanism was still realizable for the system with a larger driving force; it also confirmed that the frequency response range of the system was 35 Hz, which will be enough for responding to the control commands of human operators. As well, it is verified that an operator was able to sense both the resistant and assistant force, and to regulate the control force accordingly to perform the desired movement. This performance makes the system much suitable to provide bilateral arm coordinated training for hemiplegic patients.

However, the required input torque was still larger than the reaction torque in the slave terminal. This was mainly caused by the gear box efficiency and the amplified unloaded and inertial torques of the motors. Thus, gear boxes and motors with high working efficiency are preferred. As for the relationship between the two terminal forces, it can be modulated by matching the lengths of the two terminal force arms. If a significantly large torque is required for the actuator on the slave side, a longer force arm in the master terminal can be considered in order to reduce the burden on the operator. With regards to the reverse case, a shorter force arm on the master terminal or a longer force arm in the slave terminal can be designed to amplify a small reaction force and make it suitable for a human operator. On the other hand, if it is difficult to change the lengths of the force arms due to restrictions on working space, the relationship between the two terminal forces can be adjusted by using different motors or matching the gear ratios of the gear boxes. That is, the magnitudes of the two terminal torques in the balanced state are matched directly in view of the expected force relation.

In this paper, two terminals were controlled to implement mirror-image movements. Depending on different applications, different movement relationship of the two terminals can be achieved by attaching a scale coefficient for the velocities/positions between the two terminals, then making the movement of the slave terminal within the controllable movement range of the human operator. However, the PID parameters of the motion tracking controller should be regulated accordingly. More experiments should be performed to verify this capability.

## 5. Conclusions

The proposed sensing mechanism has several characteristics that make it suitable for application to a robot development. First, the system realizes bilateral force sensing without a mechanical force sensor. Second, the system achieves master-slave motion tracking for the both control directions. Third, the relative position between the master and slave units can be adjusted thanks to the wired connection between the two motors. These advantages give this new sensing mechanism great potential in applications to the fields of rehabilitation, minimally invasive surgery, manipulation, and so on. In particularly, the features of force sensing and bidirectional controllability are very desirable in bimanual cooperative rehabilitation training systems. However, system configuration should be improved for different application studies in our future work.
